# Evaluation of an experimental setup to analyse the intermaxillary relation in surfers

**DOI:** 10.1080/07853890.2021.1897318

**Published:** 2021-09-28

**Authors:** F. Santos, P. Cebola, S. Félix, C. Godinho, J. Rua, A. Dias, A. Almeida

**Affiliations:** a Instituto Universitário Egas Moniz (IUEM), Egas Moniz Cooperativa de Ensino Superior, Caparica, Almada, Portugal; b Centro de Investigação Interdisciplinar Egas Moniz (CiiEM), Egas Moniz Cooperativa de Ensino Superior, Caparica, Portugal; c KinesioLab, Instituto Piaget, Campus de Almada, Portugal

## Abstract

**Introduction:**

There is growing research regarding the influence of intra oral devices over sportive performance [1,2]. There is however an unclear definition of the intermaxillary relation during athletes activity and its influence upon the performance. This theme is absent across the scientific research and should be the starting key point on this kind of study. Our goal is to create and assess an experimental setup to determine the intermaxillary relation in surfers during take off - getting up on the surfboard.

**Materials and methods:**

Ten male adults free surfers were invited for this study. We applied a standard questionnaire about intermaxillary relation during sports. Two possible setups were tested in terms of distance to capture the masticatory muscles in video of 5 take offs; we used a Cannon®️ 77 D on a tripod first at a distance of 1 m from a white tape placed on the floor perpendicularly to the body of the surfer facing down, where both hands were placed while he was laying on the ground ready for take off. Then we gave them a signal to execute the takeoff and all was recorded on video for posterior slow motion analysis. The exact same procedure was done a second time for a distance of 2 metres. This case series study was approved by the Egas Moniz Ethical Committee and each patient signed previously an informed consent. All the assumptions of the Helsinki Declaration have been fulfilled.

**Results:**

Comparing the 2 setups, it was unclear, in terms of image analysis, the setup with the 1 m distance, becoming clear, in terms of muscle contraction, the 2 metres distance. Even so, the comparison between the standard questionnaire about intermaxillary relation and the video captured is highly variable and somewhat confusing, presenting some analysis limitations, giving us a clear notion that we need another mean of instrumental analysis about the intermaxillary position of the surfers.

**Discussion and conclusions:**

It is clear to us the high variability of the intermaxillary position of athletes during take off as the lack of self perception on their practice. It is mandatory to design a setup to have an instrumental analysis like electromyography to confirm masticatory muscle contraction and validate the possible influence or relation between intermaxillary relation and sportive performance.
